# Variability of the lower limb symmetry index associated with the gait parameters in the overweight adult population with flatfoot: a case-control study

**DOI:** 10.3389/fbioe.2023.1189309

**Published:** 2023-06-14

**Authors:** Israel Casado-Hernández, Ricardo Becerro-de-Bengoa-Vallejo, Marta Losa-Iglesias, Juan Gómez-Salgado, Daniel López-López, Javier Bayod

**Affiliations:** ^1^ Faculty of Nursing, Physiotherapy and Podiatry, Complutense University of Madrid, Madrid, Spain; ^2^ Faculty of Health Sciences, Universidad Rey Juan Carlos, Alcorcón, Spain; ^3^ Department of Sociology, Social Work and Public Health, Faculty of Labour Sciences, University of Huelva, Huelva, Spain; ^4^ Safety and Health Postgraduate Programme, Universidad Espíritu Santo, Guayaquil, Ecuador; ^5^ Research, Health and Podiatry Group, Department of Health Sciences, Faculty of Nursing and Podiatry, Industrial Campus of Ferrol, Universidade da Coruña, Ferrol, Spain; ^6^ Applied Mechanics and Bioengineering Group (AMB), Aragon Institute of Engineering Research (I3A), Universidad de Zaragoza, Zaragoza, Aragon, Spain

**Keywords:** adult flatfoot, gait patterns, musculoskeletal disorders, instability, lower extremity

## Abstract

**Background:** Adult acquired flatfoot is characterized by a medial arch collapse during monopodal support in the stance phase, developing eversion of the calcaneus and abduction of the forefoot linked to the hindfoot. The purpose of our research was to analyze the dynamic symmetry index in the lower limbs comparing patients with flatfoot and normal foot.

**Methods:** A case-control study was carried out with a sample of 62 participants divided into two groups consisting of 31 participants were overweight with bilateral flatfoot and 31 participants with healthy feet. A portable plantar pressure platform with piezoresistive sensors was used to measure the load symmetry index in the lower limbs in the foot areas and gait phases.

**Results**: Gait pattern analysis showed statistically significant differences in the symmetry index for lateral load (*p* = 0.004), the initial contact phase (*p* = 0.025) and the forefoot phase (*p* < 0.001).

**Conclusion:** The adults were overweight with bilateral flatfoot evidenced alterations in the symmetry index in the lateral load and in the initial contact and flatfoot contact phases, showing greater instability in overweight adult flatfoot compared to the people with normal feet.

## 1 Introduction

Flatfoot is a common foot disorder with a high prevalence in middle-aged women (Flores et al., 2019). Subjects that suffer flatfoot are characterized by biomechanical disturbances, foot and lower limb pains, bone malalignment and muscle alterations (Tahmasebi et al., 2015).

Adult acquired flatfoot is characterized by a medial arch collapse during monopodal support in the stance phase, developing eversion of calcaneus, and abduction of the forefoot linked to the hindfoot (Flores et al., 2019). The posterior tibialis tendon (PTT) is the main dynamic medial arch stabilizer avoiding arch collapse, and the spring ligament is the main static medial arch stabilizer (Mengiardi et al., 2005; 2016; Orr and Nunley, 2013). The PTT is degenerated in flatfoot, and the chronicity of the injury produces a tendon dysfunction and soft tissue abnormalities in the plantar and posteromedial foot areas (Guelfi et al., 2017; Easley and Harston, 2018). Flatfoot stability is weak due to the incorrect alignment of the ankle, knee and hip joints and is more unstable compared with normal foot because the forces applied on ligaments are reduced (Tahmasebi et al., 2015). Although flatfoot is associated with an unstable condition, the muscle activation of the tibialis anterior, peroneus lateralis longus, gastrocnemius and soleus during a transition from double to single leg stance does not differ from normal foot (Koshino et al., 2020). Normal foot gait patterns are characterized by normal subtalar and midtarsal joint pronation during the initial ground contact and total foot contact gait patterns. The proper foot function is to absorb the shock with the ground and provide stability during the gait (Resende et al., 2019).

Foot instability has a considerable influence on the quality of life of the people affected and it is greater in women than men (López López et al., 2016; López-López et al., 2018). Moreover, metabolic disorders, such as excessive body weight could lead to a collapse of the arch structure and incremental plantar loading, increasing the risk of foot injury and too the type II diabetes, have a negative impact on the foot health quality of life compared with subjects with type I diabetes (Palomo-López et al., 2019; Cen et al., 2020).

Baropodometry is a useful tool for analyzing the system that ensures postural control, balance and stability in the human body (Moffa et al., 2020) and maintains the center of body mass inside the foot contact area (Takakusaki, 2017). Postural stability means the ability to control the human body in any situation, and the main function is to maintain an upright posture downplaying the sway of the center of pressure (Woollacott and Shumway-Cook, 2002). Postural balance and body sway can be examined using a baropodometric plate (De Bengoa Vallejo et al., 2011; Becerro-de-Bengoa-Vallejo et al., 2014; Rodríguez-Sanz et al., 2018).

It is a fact that subjects with flatfoot have a high falling risk because of kinematic disorders in the gait pattern besides joint hypermobility over the terminal and mid stance phases of the gait (Awale et al., 2017; Phan et al., 2021).

Based on these antecedents, our hypothesis was that overweight adult patients with adult flatfoot would be more unstable due to postural asymmetry that would produce variability in the center of pressure, increasing contact area compared to normal foot subjects. Therefore, this a novel investigation which aimed to analyze the dynamic symmetry index in the lower limbs comparing people with flatfoot and normal foot.

## 2 Methods

### 2.1 Design and sample

A convenience sample comprising 62 participants (7 men and 55 women) was recruited based on the exclusion and inclusion criteria. The recruited subjects’ mean age was 23.48 years old, and the age range was between the 19 and 34 years old. The participants in the study were recruited employing a consecutive non-random design. The sample was divided into two groups, the case group was composed of participants were overweight with bilateral flatfoot and a total of 31 participants were chosen, and the other group, the control group, consisted of 31 participants characterized by healthy feet.

All the participants involved in the research had to meet the inclusion and exclusion criteria requirements. The inclusion criteria for the participants were overweight flatfoot group were: 1) to be between 18 and 64 years old, 2) to be healthy adults without musculoskeletal disturbances, foot disorders and illness, or outstanding common health diseases, 3) not to have any lower limb surgical history, 4) to have bilateral flatfoot, 5) to have a positive navicular drop test, and 6) to be able to comprehend and follow the research instructions. The exclusion criteria for the participants were overweight flatfoot group were: 1) to be younger than 18 and older than 65 years old, 2) to have any kind of foot pain or disturbance, 3) to take medication or receive treatment that could influence the data collection process, 4) to be pregnant or breastfeeding, 5) to suffer any musculoskeletal disorder or neurological disease, 6) not to have flatfoot, 7) to have a navicular drop test measurement of less than 9 mm, and 8) to reject or not understand the guidelines to take part in the research.

Regarding the control group, the inclusion criteria were: 1) to be older than 18 and younger than 64 years old, 2) to be healthy adults without musculoskeletal alterations, pain in the foot or significant common health diseases, 3) not to have any lower limb trauma or surgery, 4) to have bilateral neutral foot, 5) to agree to and understand the written informed consent and complete the project stages. The exclusion criteria were: 1) to be less than 18 or more than 65 years old, 2) to have any relevant foot illness, 3) to take medication or receive treatment that could influence the data collection process, 4) to be pregnant or breastfeeding, 5) to suffer any musculoskeletal disorder or neurological disease, 6) to reject or not understand the guidelines to take part in the research.

### 2.2 Procedure

The research was carried out by a specialist in podiatry in biomechanical performance assessment with more than 15 years’ experience. The first thing the podiatrist did was to interview the participants and record the clinical characteristics and global health following the same protocol for all the participants. Then each participant had to take off their shoes and socks, and the podiatrist checked and took the anthropometric measurements that consisted of height, weight and body mass index (BMI). These measurements were recorded with the participant wearing light clothes and barefoot and were analyzed using Quetelet´s equation for BMI = weight/height^2^ (Macdonald, 1986).

The navicular drop test (NDT) was used to discriminate the case group from the control group. First, subjects had to stand barefoot in a relaxed position on the floor, subsequently, the navicular tuberosity was marked with a pencil by the podiatrist. Next, inversion and eversion movements were performed by the podiatrist to neutralize the subtalar joint. Once the subtalar joint had been neutralized, the participant stood still in a non-weightbearing foot position and the distance between the tuberosity of the navicular bone from the floor was measured. This first measurement was measured with a ruler and recorded in millimeters. Secondly, the participant changed the non-weightbearing to a weightbearing standing foot position and the podiatrist measured the distance between the tuberosity of the navicular bone and the floor. This second measurement was measured with a ruler and recorded in millimeters. Finally, a positive NDT was the result of the difference between these heights measurements being greater than 10 mm. The measurements were repeated three times on each participant and the mean was recorded (Spörndly-Nees et al., 2011; Zuil-Escobar et al., 2018).

A portable plantar pressure platform with resistive sensors was used to analyze the dynamic symmetry in all the participants (Neo-Plate, Herbitas. Spain). This portable plantar pressure platform is a reliable device for foot diagnosis (Painceira-Villar et al., 2021). Following the protocol of Becerro-de-Bengoa-Vallejo et al., the dynamic data were recorded at a constant walking speed and included foot surface areas and load percentages in initial contact, flatfoot and forefoot contact phases collected in the right and left lower limbs and in the flatfoot arch and normal arch of each participant (Vallejo et al., 2013).

This podiatry protocol consists in barefoot subjects walking at a constant, self-selected speed. Practice trials were performed to determine a walking speed for each subject and to facilitate platform striking with the total foot. After the adaptation of the testing methods was completed, data were collected. A trial was thought valid when a heel-strike–toe-off pattern was performed and the walking speed was constant (De Cock et al., 2006). Trials that did not meet each of these criteria were not used for subsequent analysis. A 2-step procedure in which plantar pressure data were analyzed on the following step of each foot was used for each walking trial, and data for 6 steps with each foot were recorded per session from each participant. The mean of 6 trials per foot was used for statistical testing.

### 2.3 Plantar pressure platform analysis

The baropodometric platform was composed of resistive sensors with dual amplifier, and an automatic multipoint calibration was performed, as required for correct use by the manufacturer before beginning measurement. The portable plantar pressure platform was 40 × 40 cm, with a flat surface 8 mm thick; the platform weighed 4 kg and comprised 4,096 resistive sensors. Each platform sensor measurement was to the nearest 0.01 kPa and the vertical force was recorded at frequencies between 100 and 500 Hz. The platform was connected by an USB interface unit to a personal laptop and the acquired data were analyzed with Neo-Plate software (Herbitas, Foios-Valencia, Spain).

For this study, the symmetry index (Supporting Material) was applied, defined as the comparison of the load for both lower limbs using the following equation $SI=\frac{Xr-Xi}{0.5\left|Xr\right|+\left|Xi\right|}.100$ , xr is the variable determined for the right limb and xi the variable determined for the left limb.

This is a procedure of percentage measurement of the differences involving the kinematic and kinetic values on the left and right lower limbs during human gait and is the technique most usually employed and cited in investigations on walking symmetry (Błazkiewicz et al., 2014).

The parameters of symmetry index = 0 reflects full symmetry, whereas symmetry index ≥100% indicates asymmetry (Sadeghi et al., 2000).

Dynamic pressure maps were also created for body weight on the lower limb variables which included: 1) Anterior load, the measured load from the midtarsal joint to the forefoot; 2) Posterior Load, the measured load from the midtarsal joint to the rearfoot; 3) Medial Load, the measured load from the bisector from the second toe to the medial foot area; 4) Lateral Load, the measured load from the bisector from the second toe to the lateral foot area; 5) Initial Contact Phase (ICP), the measured load during the first initial floor contact with the calcaneus bone and continuing until the contralateral foot was lifted for swinging; 6) Flatfoot Contact Phase (FFCP), the measured total contact of the foot; and finally 7), Forefoot Phase (FFP), measured from the final stance to the toe off gait phase. All the foot variables measured were compared and analyzed by percentages and meters per second.

### 2.4 Sample size calculation

The sample size was calculated with G* Power 3.1.9.3 software (Heinrich-Heine-Universität Düsseldorf, Germany) and it tested the correlation between two paired means regarding correspondence with a Spearman correlation coefficient of 0.40 and a 95% confidence interval (CI) for a two-tailed test, an *a* error of 0.05 and an estimated analysis power of 80% (error *ß* = 20%). The minimum sample size consisted of 62 participants (31 per group).

### 2.5 Ethical and legal considerations

This study was carried out from May to September 2022 following all the Strengthening the Reporting of Observational Studies in Epidemiology (STROBE) guidelines (Vandenbroucke et al., 2014). The study was certified by an ethics committee and all the requirements were considered following the ethical standards for human research explained in the Declaration of Helsinki (Shrestha and Dunn, 2020). Subjects were recruited by a human movement laboratory at the Universidade da A Coruña, located in the city of Ferrol (Spain) and participated in the project with record number PID 2019-108009RB-I00 which received the approval from the Research Ethics Committee at the University of A Coruña, Spain; Identification document number 2019–0017; date: 6 November 2019.

### 2.6 Statistical analysis

The normality of the dynamic symmetry index variables was analyzed and checked with the Kolmogorov-Smirnov test, with statistical significance of *p* > 0.05. The results of the independent Student’s t-tests were used to determine if the data were normally distributed and parametric statistical tests were most appropriate. The non-parametric Mann-Whitney “U” test was used to consider discrepancies between the two groups with or without adult flatfoot.

The independent variables are shown as mean, ranges of minimum to maximum and standard deviation (SD) values for the descriptive data analysis. Concerning the categorical variables, they were presented as percentages and absolute values. The Neo-Plate software version for Windows was used to acquire the dynamic symmetry index produced for each foot with or without adult flatfoot.

The analysis of the outcomes was performed with the IBM SPSS Statistics 27.0.01.0 package for windows (Armonk, NY, USA). For all the analyses, significance was established at *p* < 0.05 with a 95% confidence interval.

## 3 Results

### 3.1 Sociodemographic data

A total sample of 62 subjects, between 19 and 34 years old with a mean age ± SD of 23.48 ± 5.46 years, completed all the research. Most voluntary participants were overweight with a total sample weight of 71.26 ± 14.58 kg and a total sample Body Mass Index (BMI) of 26.51 ± 5.61 kg/m^2^, both showing statistically significant differences between groups (*p* < 0.001). The main descriptive characteristics of all the subjects are presented in Table 1 stratified by groups with or without bilateral adult flatfoot, and no statistically significant differences are shown.

**TABLE 1:** Main characteristics of total sample with or without bilateral adult flatfoot.

Characteristics	Total sample (*n* = 62)	Case group (*n* = 31)	Control group (*n* = 31)	*p*-value
	Mean ± SD (range)	Mean ± SD (range)	Mean ± SD (range)	
Age (years)	23.48 ± 5.46 (19–34)	23.87 ± 4.23 (19–34)	23.10 ± 4.21 (19–34)	0.097†
Weight (kg)	71.26 ± 14.58 (48–98)	77.36 ± 15.10 (56–98)	65.16 ± 11.28 (48–89)	< 0.001†
Height (cm)	164.69 ± 7.44 (152–185)	163.13 ± 6.54 (152–175)	166.26 ± 8.05 (155–185)	0.138†
BMI (kg/m^2^)	26.51 ± 5.61 (19.00–39.26)	29.25 ± 6.29 (25.01–39.26)	23.76 ± 2.97 (19.00–30.48)	< 0.001†
Sex, male/female (%)	7/55 (11.3/88.7)	4/27 (12.9/87.41)	3/28 (9.7/90.3)	1.000 ‡
Foot Size (N)	38.84 ± 2.15 (36–46)	38.55 ± 1.71 (36–42)	39.12 ± 2.51 (36–46)	0.436†

**Abbreviations:** Kg, Kilogram; Cm, Centimeter; m^2^ Square meter; % Percentage; SD, standard deviation; N, Number. † Mann-Whitney *U* test was used. ‡ Fisher exact test was used. In all the analyses, *p* < 0.05 (with a 95% confidence interval) was considered statistically significant (bold).

### 3.2 Main outcome measures data

The main outcome measurements of the symmetry index analysis are described in Table 2. Statistically significant differences were shown in the medial load in the lower right limb variable between groups: the case group 51.27% ± 5.24 (40.60—56.50) and the control group 54.71% ± 4.04 (49.60—61.80) with a *p* = 0.028; and the lateral load in the lower right limb variable between groups: the case group 48.73% ± 5.24 (43.50—59.40) and the control group 45.29% ± 4.04 (38.20—50.40) with a *p* = 0.028. Besides, the symmetry index lateral load variable displayed statistically significant differences between the case group 87.04 ± 5.76 (76.70—99.50) and the control group 84.40 ± 5.90 (72.10—97.70) with a *p*-value 0.004; as well as the symmetry index FFP variable in the case group 81.08 ± 8.58 (64.00—90.70) and the control group 90.31 ± 9.29 (72.10—97.70) with a *p*-value < 0.001; and, finally, the symmetry index ICP variable in the case group 81.69 ± 15.36 (51.90—96.50) and the control group 87.63 ± 14.07 (53.00—100.00) with a *p*-value 0.025. The remaining variables did not display statistically significant differences between groups.

**TABLE 2:** Main outcome measurements of symmetry index analysis of total sample with or without bilateral adult flatfoot.

Characteristics	Total sample (*n* = 62)	Case group (*n* = 31)	Control group (*n* = 31)		*p*-value
	Mean ± SD (range)	Mean ± SD (range)	Mean ± SD (range)		
Anterior load in the lower left limb (%)	48.73 ± 3.37 (41.80—53.70)	49.24 ± 3.37 (42.80—53.70)		48.23 ± 3.34 (41.80—53.20)	0.355 †
Posterior load in the lower left limb (%)	51.27 ± 3.37 (46.30—58.20)	50.76 ± 3.37 (46.30—57.20)		51.76 ± 3.34 (46.80—58.20)	0.355 †
Medial load in the lower left limb (%)	45.18 ± 4.10 (36.70—52.80)	44.04 ± 4.11 (36.70—48.70)		46.33 ± 3.83 (40.80—52.80)	0.156 †
Lateral load in the lower left limb (%)	54.83 ± 4.11 (47.20—63.30)	55.96 ± 4.11 (51.30—63.30)		53.71 ± 3.85 (47.20—59.20)	0.156 †
Left foot FFP (ms)	281.63 ± 73.52 (162.70—442.00)	294.04 ± 78.27 (176.20—432.60)		269.23 ± 67.43 (162.70—442.00)	0.183 †
Left foot FFCP (ms)	389.02 ± 84.93 (259.20—605.60)	393.95 ± 103.78 (283.20—605.60)		384.10 ± 62.03 (259.20—465.60)	0.827 †
Left foot ICP (ms)	88.65 ± 32.14 (43.70—149.40)	82.83 ± 32.10 (43.70—127.60)		94.48 ± 31.62 (44.60—149.40)	0.148 †
Anterior load in the lower right limb (%)	47.58 ± 4.36 (40.30—54.90)	48.25 ± 4.36 (41.20—54.90)		46.90 ± 3.66 (40.30—52.20)	0.233†
Posterior load in the lower right limb (%)	52.42 ± 4.36 (45.10—58.80)	51.75 ± 4.93 (45.10—58.80)		53.10 ± 3.66 (47.80—59.70)	0.233†
Medial load in the lower right limb (%)	52.99 ± 4.96 (40.60—61.80)	51.27 ± 5.24 (40.60—56.50)		54.71 ± 4.04 (49.60—61.80)	0.028†
Lateral load in the lower right limb (%)	47.58 ± 4.36 (38.20—59.40)	48.73 ± 5.24 (43.50—59.40)		45.29 ± 4.04 (38.20—50.40)	0.028†
Right foot FFP (ms)	271.83 ± 97.73 (165.80—567.50)	279.46 ± 97.73 (193.40—567.50)		264.20 ± 67.13 (165.80—405.00)	0.597 †
Right foot FFCP (ms)	384.92 ± 109.32 (34.15—544.00)	402.93 ± 103.50 (236.80—544.00)		366.91 ± 113.09 (34.15—513.20)	0.095†
Right foot ICP (ms)	89.99 ± 31.58 (42.80—141.40)	90.13 ± 30.61 (43.80—141.40)		89.85 ± 33.03 (42.80—137.60)	0.882 †
Symmetry index anterior load (%)	94.44 ± 4.26 (80.70—99.10)	94.98 ± 3.24 (89.10—99.00)		93.90 ± 5.36 (80.70—99.10)	0.961 †
Symmetry index posterior load (%)	94.95 ± 4.00 (82.00—99.00)	95.54 ± 2.44 (91.40—98.80)		94.37 ± 5.08 (82.00—99.00)	0.762†
Symmetry index medial load (%)	85.43 ± 5.42 (75.90—99.40)	86.15 ± 5.46 (76.50—99.40)		84.70 ± 5.36 (75.90—97.90)	0.070 †
Symmetry index lateral load (%)	85.72 ± 5.93 (72.10—99.50)	87.04 ± 5.76 (76.70—99.50)		84.40 ± 5.90 (72.10—97.70)	0.004 †
Symmetry index FFP (%)	85.69 ± 10.01 (64.00—99.90)	81.08 ± 8.58 (64.00—90.70)		90.31 ± 9.29 (73.50—99.90)	< 0.001 †
Symmetry index FFCP (%)	89.62 ± 8.30 (69.10—99.30)	87.60 ± 9.48 (69.10—98.50)		91.63 ± 6.45 (79.60—99.30)	0.082†
Symmetry index ICP (%)	84.66 ± 14.91 (51.90—100.00)	81.69 ± 15.36 (51.90—96.50)		87.63 ± 14.07 (53.00—100.00)	0.025 †

**Abbreviations:** ICP, initial contact phase; FFCP, forefoot contact phase; FFP, flatfoot phase. Ms, meters per second; % percentage; SD, standard deviation; † Mann-Whitney *U* test was used. In all the analyses, *p* < 0.05 (with a 95% confidence interval) was considered statistically significant (bold).

## 4 Discussion

The purpose of this research was to analyze the variability of the foot load symmetry index in dynamic situations between adults participants were overweight with bilateral flatfoot and normal foot subjects using a plantar pressure platform. The research protocol used to acquire the data variables was the same as in the previous study by Becerro de Bengoa et al. (Vallejo et al., 2013)

Vuurberg et al. performed a meta-analysis to identify the intrinsic factors involved in ankle instability and ankle lateral sprains. The main findings reported that subjects with overweight developed chronic ankle instability and subjects with higher BMI only had ankle sprains (Vuurberg et al., 2019). According to our results, all the patients with flatfoot were overweight 77.36 Kg ± 15.10 Kg and had a high BMI 29.25 ± 6.29 kg/m^2^. Our results showed statistically significant differences in the symmetry index lateral load between the case group and the control group (*p* = 0.004), with the adults with flatfoot being more unstable in the foot lateral load coinciding with the meta-analysis regarding ankle lateral instability.

We also found similarities in our findings with previous research carried out by Ko et al., showing that overweight adults had a decreased ankle range of motion in kinematic gait patterns in antero-posterior and medial-lateral planes while walking at normal and faster speeds (Ko et al., 2010). According to our research, the medial and lateral load in the lower right limb showed a statistically significant result (*p* = 0.028), with similar results to the Ko et al. research where that they did not mention if the patients with overweight had flatfoot and assumed that all the subjects presented symmetry during walking analysis. Another important finding in the Ko et al. research was the stride width spatiotemporal variable that showed statistically significant differences between the normal and overweight patients. Considering these findings, the postural transition in overweight adult patients from the stance position to the initial contact phase produces alterations in medio-lateral movements increasing the center of pressure speed but decreasing in antero-posterior movements compared to the control group (Cau et al., 2014). This finding supports the alterations in the symmetry index lateral load in our research, with the stride width being greater in overweight patients and the load increased in the lateral area of the foot.

According to previous research performed by de Castro et al., overweight patients were not able to improve their stability in mediolateral ground reaction forces compared to the normal foot group. In addition, they reported that the higher peak plantar pressure was distributed in the central rearfoot, in the lateral midfoot area and in the lateral and central forefoot area. In addition, the lateral foot area was the most loaded when walking (de Castro et al., 2014). These conclusions support our findings, according to the symmetry index, in the initial and flatfoot contact phases with statistically significant differences, but we did not find statistically significant differences in the forefoot contact phase. However, this can be explained based on the de Castro et al. study, because they stated that the forefoot area had the lowest load in overweight patients during walking, protecting the hallux.

We should mention some limitations in our research as a longitudinal evaluation of multifactorial interactions among intrinsic risk aspects of adult flatfoot (age, arch height, body mass index and sex) could be considered to improve the power of this investigation, and could have helped to determine the association that is not known about the different mechanisms involved. According to our results, we only found statistically significant differences in the medial and lateral load in the lower right limb between groups, in future research the lower limb dominance should be considered as previous studies have shown that a greater volume is developed in the dominant leg in healthy individuals (Dylke et al., 2012). In addition, another limitation in our results was to analyze the effects of electromyography and kinematics parameters in biomechanical lower limb gait phases that were not recorded, because of this, no statistically significant differences were found between groups in the symmetry index FFCP. For future research these parameters signal the need to evaluate the impact of obesity and the importance of continuing to study instability in the adult population with flatfoot and the effect of dominant vs non-dominant side limb on quality of life.

## 5 Conclusion

The adults were overweight with bilateral flatfoot evidenced symmetry alterations in the symmetry index in the lateral load and in the initial contact and flatfoot contact phases, showing higher instability in overweight adult flatfoot compared to normal foot subjects.

## Data Availability

The raw data supporting the conclusion of this article will be made available by the authors, without undue reservation. Requests can be made to daniellopez@udc.gal.
